# Detecting biased validation of predictive models in the positive-unlabeled setting: disease gene prioritization case study

**DOI:** 10.1093/bioadv/vbad128

**Published:** 2023-09-14

**Authors:** Ivan Molotkov, Mykyta Artomov

**Affiliations:** The Steve and Cindy Rasmussen Institute for Genomic Medicine, Nationwide Children’s Hospital, Columbus, OH, United States; Department of Pediatrics, The Ohio State University, Columbus, OH, United States; ITMO University, Saint Petersburg, Russia; The Steve and Cindy Rasmussen Institute for Genomic Medicine, Nationwide Children’s Hospital, Columbus, OH, United States; Department of Pediatrics, The Ohio State University, Columbus, OH, United States

## Abstract

**Motivation:**

Positive-unlabeled data consists of points with either positive or unknown labels. It is widespread in medical, genetic, and biological settings, creating a high demand for predictive positive-unlabeled models. The performance of such models is usually estimated using validation sets, assumed to be selected completely at random (SCAR) from known positive examples. For certain metrics, this assumption enables unbiased performance estimation when treating positive-unlabeled data as positive/negative. However, the SCAR assumption is often adopted without proper justifications, simply for the sake of convenience.

**Results:**

We provide an algorithm that under the weak assumptions of a lower bound on the number of positive examples can test for the violation of the SCAR assumption. Applying it to the problem of gene prioritization for complex genetic traits, we illustrate that the SCAR assumption is often violated there, causing the inflation of performance estimates, which we refer to as validation bias. We estimate the potential impact of validation bias on performance estimation. Our analysis reveals that validation bias is widespread in gene prioritization data and can significantly overestimate the performance of models. This finding elucidates the discrepancy between the reported good performance of models and their limited practical applications.

**Availability and implementation:**

Python code with examples of application of the validation bias detection algorithm is available at github.com/ArtomovLab/ValidationBias.

## 1 Introduction

Positive-unlabeled (PU) data consists of objects that are labeled positive and objects that are unlabeled. The latter could be either positive or negative (Elkan and Noto 2008). PU data naturally arises in many scientific and practical settings—healthcare records, where an absence of diagnosis does not necessarily mean an absence of the corresponding phenotype (Zuluaga *et al.* 2011); fake online reviews detection, where some reviews can be manually labeled to be fake, while the vast majority will remain unlabeled (Li *et al.* 2014); and disease gene identification, where it might be possible to find a pathway through which a gene influences the disease, but currently it is impossible in practice to prove that no such pathway exists (Kolosov *et al.* 2021).

PU data can be contrasted with standard data that is usually used for binary classification, where all positive and negative labels are known. Without making any assumptions, accurate performance estimation of a binary classifier using PU data is not possible, because different choices of positive labels for the same ground truth positive-negative data will yield different performance estimates. Thus, the limited knowledge of the true labels creates additional challenges, requiring making assumptions about the underlying data structure and labeling process to effectively train and accurately validate models.

The most widely used is the assumption that positive examples have the same probability to be labeled—the so-called Selected Completely At Random (SCAR) assumption. When it holds, metrics, such as recall or average rank of a positive example can be estimated directly by considering unlabeled data as negative. Additionally, unbiased estimates of areas under ROC or precision–recall curves require knowledge of class priors (Claesen *et al.* 2015, Ramola *et al.* 2019). This information can either be obtained from prior domain knowledge, or in some cases it can be estimated from data (Christoffel *et al.* 2016, Jain *et al.* 2016, Ramaswamy *et al.* 2016).

**Figure 1.**
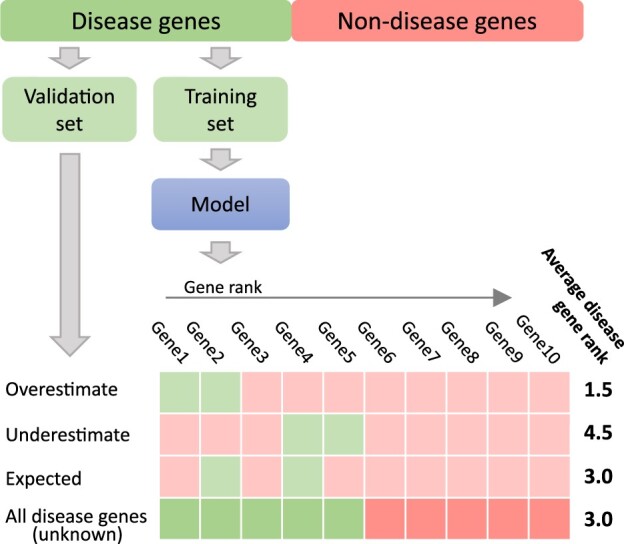
Visualized process of training and evaluating a gene prioritization model. Genes for training and validations are assumed to be taken from the set of all the true disease genes. The resulting model is then evaluated using a validation set. Such estimation can over- or under-estimate the true model performance if the validation set is not representative of all the disease genes.

The problem of disease gene identification has become a playground for testing new PU-specific approaches (Mordelet and Vert 2011, Yang *et al.* 2012, 2014, Nikdelfaz and Jalili 2018, Vasighizaker and Jalili 2018, Arabfard *et al.* 2019). Dozens of gene prioritization models were developed to aid the disease gene identification problem (Zolotareva and Kleine 2019). Validation sets of positive examples of gene–disease associations are taken from the gold standard gene sets—collections of genes that are known to be related to a disease. Then, models are validated by standard binary classification performance metrics after considering genes that are not in the gold standard sets to be unrelated to a disease (Fig. 1). However, this approach can yield an unbiased estimation of the true performance of a model only under the SCAR assumption and only for certain metrics.

The SCAR assumption is usually assumed for convenience, without any justifications for it. Moreover, for the gene prioritization problem, it is strongly suspected that the SCAR assumption is systematically violated. Several explanations for this phenomenon were proposed: (i) known genes are often better studied and annotated, making it easier for models to detect them, often referred to as “knowledge bias” (Gunning and Pavlidis 2021) and (ii) known genes are detected more easily due to crosstalk effects between data sources (Börnigen *et al.* 2012). Consequently, models suffer from poor generalization performance, meaning that they overfit to the known genes and struggle to classify genes that have not been seen before. Because of this phenomenon, which we refer to as validation bias, performance estimates obtained using validation sets can be inflated. This inflation may explain the notable discrepancy between the impressive reported performance of gene prioritization tools and their limited practical utility in novel disease gene discovery (Moreau and Tranchevent 2012) and inconsistency in predictions (Zhou *et al.* 2022).

In this study, we developed a simulation-based hypothesis testing procedure that can reject the null hypothesis that the SCAR assumption holds. The only assumption that the procedure requires is the lower bound on the true number of disease genes. Alternatively, it can return the minimum true number of disease genes required for the rejection of the null hypothesis. Additionally, the procedure does not make any assumptions about the gene prioritization models that are being tested.

The validation bias detection algorithm was applied to real examples of validations of gene prioritization models. In most cases, validation bias was successfully detected. Sufficient validation bias can cause the estimated performance of a low-quality model to match an unbiased performance estimation for a perfect model. As such, the analysis of validation bias has revealed that the common approach to validation using gold standard gene sets does not accurately reflect the actual model performance.

## 2 Methods

### 2.1 Validation bias detection procedure

**Figure 2.**
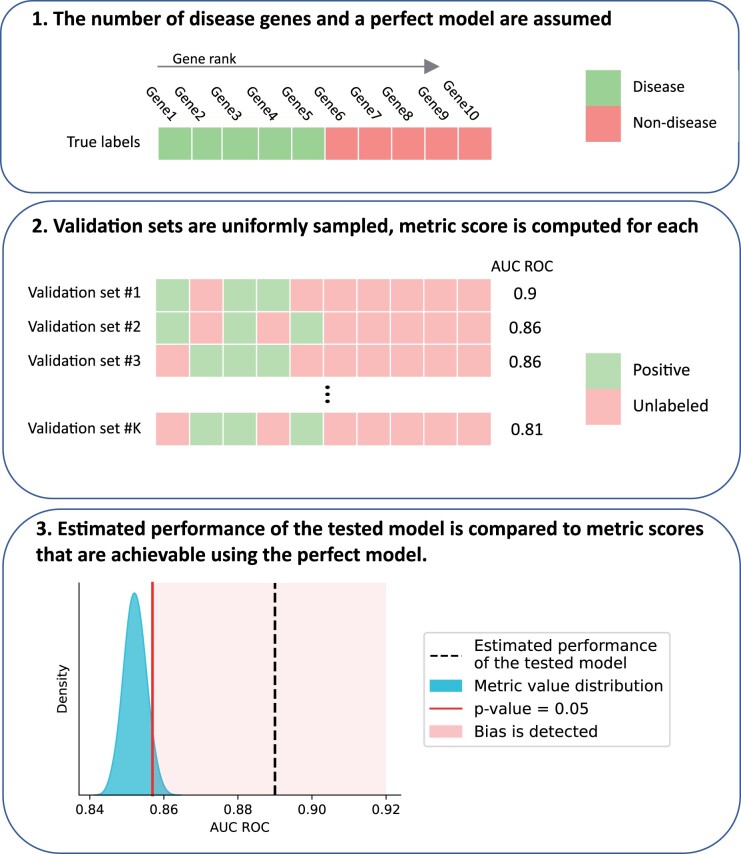
Validation bias detection procedure applied to a toy example in which the tested model’s ranking of 10 genes was estimated to have AUC ROC = 0.9 using a validation set of size 3. (1) Assume perfect model: all five disease genes are ranked higher than all non-disease ones. (2) Randomly assemble validation sets of size 3 from the disease genes. For each validation set, compute the metric value that the perfect model would receive. (3) The estimated performance of the tested model is significantly better than estimates obtained for the perfect model—thus, validation bias is detected.

The validation bias detection procedure requires information about (i) validation set size *N*, (ii) the approximate total number of the disease genes *M*, (iii) the type of ranking quality metric that was used in the study, and (iv) metric value *R* that was obtained in the validation. If raw data are given, not only the original quality metric but any user-defined metric can be computed and tested for validation bias. The resulting procedure has several steps (Fig. 2):

1. Assume a perfect prioritization model—each disease gene has a higher score than each non-disease gene.
2. Start randomly sampling validation sets of size *N* from a set of *M* disease genes. For each validation set, compute the resulting metric of gene prioritization quality.
3. Calculate the empirical *P*-value—a proportion of prioritization quality metric values for the perfect model that are as good or better than the estimated performance of the tested model. If it is <0.05—the tested model’s estimated performance is considered to be significantly better than the performance of a perfect model without validation bias, suggesting that significant validation bias was present during tested model validation.

Although it is assumed that all the genes for the training and validation are truly related to a disease, this assumption is not important for the validation bias detection procedure. On the contrary, allowing non-disease genes to appear in the validation set would simplify the detection of validation bias (Supplementary Materials, Effect of the validation set contamination with non-disease genes on validation bias detection, Supplementary Fig. S1).

The exact number of the disease genes is not necessary for validation bias detection. Instead, it is possible to use the lower bound—large enough so it is possible to detect validation bias but not too large to be biologically implausible (Supplementary Materials, Effect of the choice of the lower bound on the total disease genes number on the detection of validation bias, Supplementary Fig. S2).

### 2.2 Distribution of the number of positive labels in the top-*k* ranked data points

Recall@*k* represents the proportion of genes from the validation set that are in the list of top-*k* ranked genes: $\mathrm{recall}@k=\frac{\mathrm{TP}}{\mathrm{TP}+\mathrm{FN}}=\frac{\mathrm{TP}}{\mathrm{Validation}\;\mathrm{set}\;\mathrm{size}}=\mathrm{TPR}$, if we consider only top-*k* ranked genes to be positive predictions. Similarly, $\mathrm{precision}@k=\frac{\mathrm{TP}}{k}$. For these metrics, only the numerator is random and depends on a particular validation set that is used for the estimation. Thus, if we could derive the distribution of the TP under the SCAR assumption for the perfect model, we could derive *P*-values for the validation bias test analytically.

Under the assumption of the perfect model and the SCAR assumption, the generative process of TP values can be described as follows: (i) there are M disease genes in total, (ii) k of them are top-k ranked because we use the perfect model, and (iii) N of the disease genes are sampled into a validation set. Then, TP indicates how many of the sampled genes are in the top-*k*. Using this formulation, we can notice that the number of TP follows a hypergeometric distribution with the total population of M, k objects of interest and N draws.

Let us denote the cumulative distribution function of hypergeometric distribution as F(x | M,k,N), which represents the probability to get not more than x true positives by a perfect model under the SCAR assumption with validation sets of size N sampled from the total number of M disease genes. Then, the *P*-value for the validation bias test is

P(recall@k ≥ x/N)=P(precision@k ≥ x/k)=P(TP ≥ x)=1 - F(x - 1 | M,k,N).

Furthermore, the algorithm can provide a lower bound for the minimum number of disease genes necessary to refute the SCAR assumption. This lower bound can be determined as follows:

$$M_{\min}=\min\;\{M\;\in \;[k,\;\infty ]:\;(1\;-\;F(x-1\;|\;M,k,N))<\;0.05\}.$$

Thus, there is no need to make assumptions about the exact total number of disease genes. Instead, the algorithm can offer the researcher the minimum number of disease genes required to detect validation bias. Subsequently, it is up to the researcher to assess the plausibility of this lower bound.

Since the *P*-values are the same for all metrics that only depend on the TP, in simulations only recall@k is used. Also, for recall@*k*, we analytically derive the three contributors to the highest realistically achievable performance metric values: validation set contamination, true model performance, and the total number of disease genes (Supplementary Materials, Effect of the validation set contamination with non-disease genes on validation bias detection). If one can justify lowering the first two or increasing the last one—the power of the validation bias detection test would increase, therefore, it would be easier to detect validation bias.

### 2.3 Mathematical representation of validation bias

**Figure 3.**
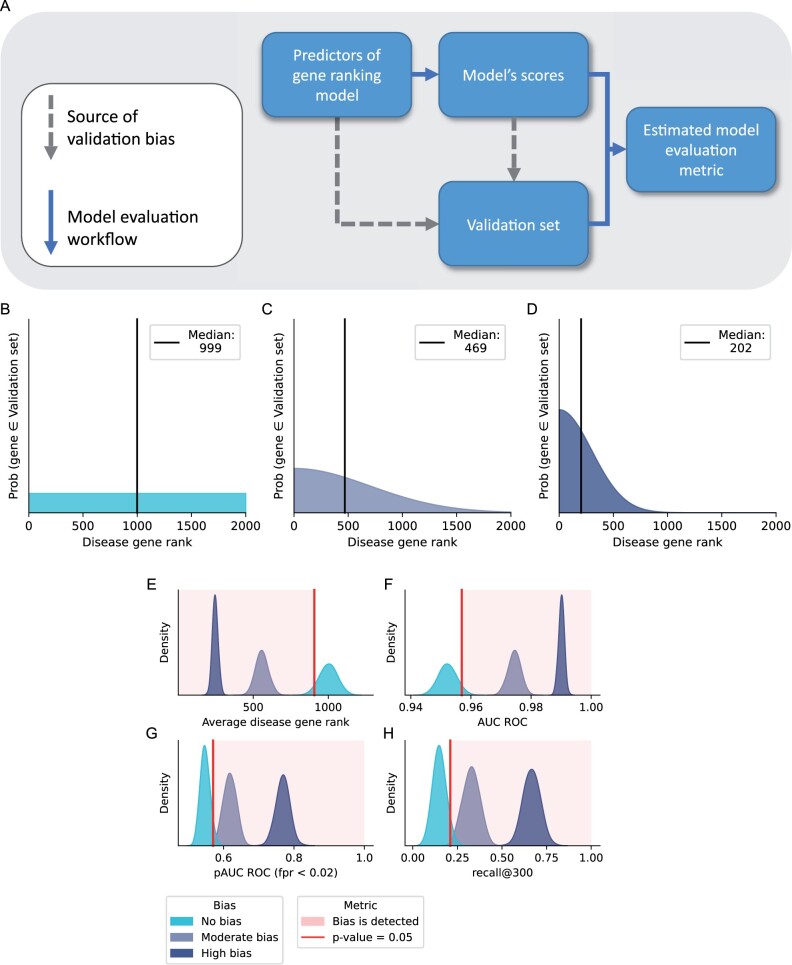
Sources of validation bias and how it affects model evaluation metrics. (A) Two potential sources of validation bias: similar gene properties are used for obtaining validation sets and model scores; validation sets are chosen with model scores in mind. (B–D) Probability distributions associated with a gene of rank *k* to be in a validation set. Ideally, we want to sample genes independently (B), but validation bias may skew distribution toward genes with lower ranks (C and D), thus causing overestimation. (E–H) Distributions of simulated model evaluation metric values as estimated from samples of validation sets of size 100 with different validation biases.

The general idea behind validation bias is that more highly ranked disease genes tend to be included in validation sets more frequently due to common confounders (Fig. 3A). We can represent this mathematically using discrete distributions over disease gene ranks. Ideally, if a validation set is assembled completely at random, this distribution will be uniform (Fig. 3B), but in the presence of validation bias, it will be skewed toward highly ranked genes (Fig. 3C and D).

In the simulations, we assumed that there are 2000 disease genes in total and defined three different discrete probability distributions over them, assigning probability based on the gene’s rank $-r\in \left\{1,\;2,\;3,\ldots ,\;2000\right\}$. Validation sets are assembled using a numpy.random.choice() function from numpy (Harris *et al.* 2020) with the probabilities specified below:

pno biasr∝1pmoderate biasr∝Nr  mean=0,std=700)phigh biasr∝Nr  mean=0,std=300).

The robustness of different evaluation metrics to the choice of the validation set size was analyzed (Supplementary Materials, Effect of the validation set size on performance estimation using different metrics, Supplementary Fig. S3).

### 2.4 Simulations of prioritization scores for gene prioritization models of different quality

Three gene prioritization models of different quality ($M_{\mathrm{perfect}},M_{\mathrm{avg}}$,$M_{\mathrm{bad}}$) were defined. The expected score of a disease gene is larger than that of a non-disease gene for all models—as such, all of them are better than random. Based on the model type ($M_{\mathrm{perfect}},\;M_{\mathrm{avg}}$,$M_{\mathrm{bad}}$), and gene type (disease and non-disease), distributions of gene scores-s were defined as:

ps  disease, Mperfect) ∝ Unif(3/4, 1)ps  nondisease, Mperfect) ∝ Unif(0, 1/4) ps  disease, Mavg) ∝ Ns  mean=1, std=0.4)ps  nondisease, Mavg) ∝ Ns  mean=0, std=0.4) ps  disease, Mbad) ∝ Ns  mean=1, std=0.6)ps  nondisease, Mbad) ∝ Ns  mean=0, std=0.6).

The resulting AUC ROC score for the perfect model is 1.00, for the average model—0.87, and for the bad model—0.71.

### 2.5 The general approach to simulations of performance estimation metric values

The simulations design that was presented in Section 2.1 and used throughout this work can be repurposed for other types of analysis. The approach provides a way to explicitly state the assumption of the validation process (such as the proportion of positively labeled data; the presence of validation bias, contamination level, and size of the validation set; model quality) and analyze how performance estimates using different metrics would respond to those assumptions.

The general approach to simulations can be formulated as follows:

1. Determine the number of positive and negative data points.
2. Establish the quality of the model: score/rank distribution for positive and negative data points.
3. Define the mechanisms of validation set assembly: validation set sizes, presence of validation bias, and contamination of validation sets with negative examples.
4. Randomly sample validation sets based on the parameters established in the previous step. For each validation set, calculate the values of performance metrics of interest.
5. Analyze the resulting distributions of model performance evaluation metric values based on the assumptions set earlier.

In general, the algorithm proposed above could help with analyzing how the tested model performance estimate diverges from simulated estimations using a certain set of assumptions, as in the validation bias detection case, or with testing the robustness of different metrics to the assumptions made in Steps (1–3).

## 3 Results

Good prioritization models should assign high scores to all disease genes, not just the ones in the validation set. Consequently, using a validation set for model evaluation is expected to result in a significant number of false positives (FPs), representing highly ranked disease genes not included in the validation set. Some of these FPs could potentially be undiscovered true disease genes. If an insufficient number of FPs is observed, it suggests that the model is only predicting validation genes and not disease genes in general, indicating the presence of validation bias.

To estimate model performance and detect validation bias, simulations can be used to determine the best quality metric value that is rarely attainable even by a perfect model without validation bias. This provides an upper bound on the estimated prioritization model quality. If the estimated quality of the tested model exceeds this upper bound—the SCAR assumption is violated, and the validation bias is present.

The more genes are involved in the disease risk, the easier it is to detect validation bias—since the corresponding required number of FPs grows accordingly (Supplementary Fig. S2). This implies that such an approach to validation bias detection is better powered for highly polygenic traits.

### 3.1 The effect of simulated validation bias on models of different levels of performance

We developed a simulation framework for scenarios where we have complete knowledge of the ground truth and can fully quantify the effects of validation bias. We assumed a disease with 2000 involved risk genes, the size of the validation gene sets of 100, and an ideal model that perfectly discriminates the disease from non-disease genes (Section 2.1). In the absence of validation bias, genes are selected into the validation sets at random with a uniform probability (Fig. 3B). Validation bias is added by assigning a higher probability to be selected into the validation set to the highly ranked genes (Fig. 3C and D), as described in Section 2.3.

First, to see the validity of the method, we assumed the perfect model with different levels of validation bias. In this setting, validation bias was detected for all metrics (Fig. 3E–H), which is to be expected. However, to test the power of the validation bias detection test, we needed to see its ability to detect validation bias for less-than-perfect models.

We simulated the results of two gene prioritization models of imperfect but known true quality (Section 2.4). For each of them, we generated 10 000 validation sets using different validation bias levels, computed the resulting metric scores for each of them, and plotted the distributions of the scores.

**Figure 4.**
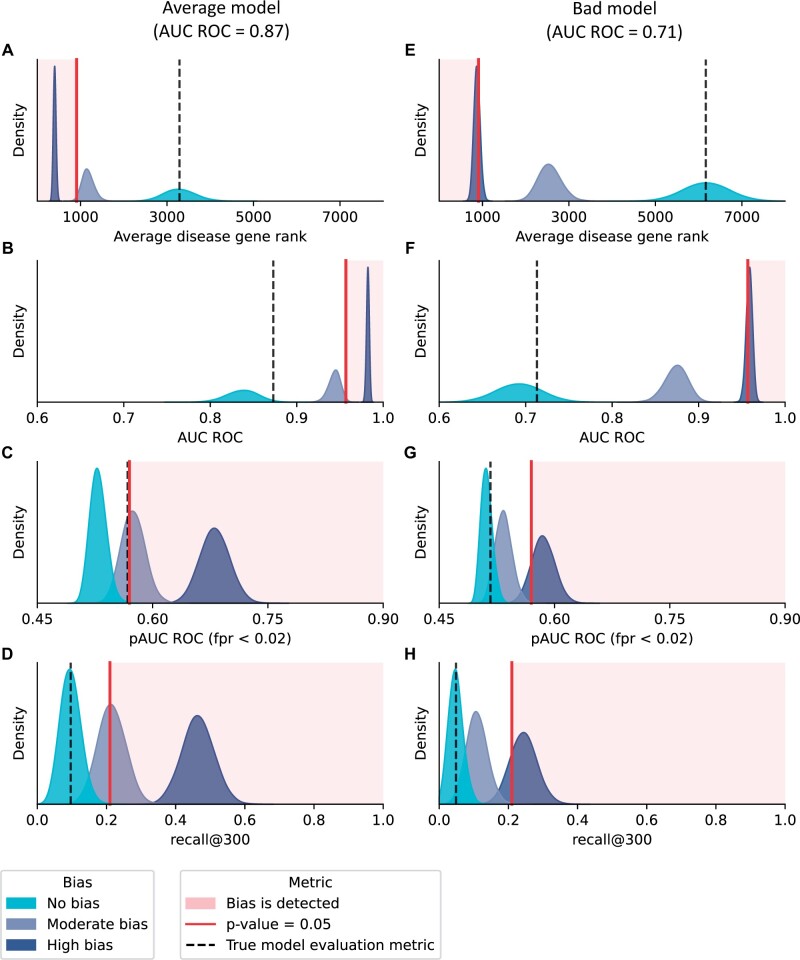
The effect of simulated validation bias on models of different levels of performance. (A–D) Distributions of simulated model evaluation metric values as estimated from samples of validation sets of size 100 with different validation biases for the average model (true AUC ROC = 0.87). (E–H) Distributions of simulated model evaluation metric values as estimated from samples of validation sets of size 100 with different validation biases for the bad model (true AUC ROC = 0.71).

The larger the validation bias, the better the observed prioritization performance is using all four quality metrics. For example, high bias was detected for both the average and the bad models (Fig. 4F–I); however, moderate bias was only detected for the average model (Fig. 4A–D). As such, validation bias is more easily detected for better models. It means that the detection of validation bias can imply either good model performance with moderate validation bias, or vice versa—poor model performance with high validation bias.

Figure 4 indicates that moderate validation bias is enough to inflate the estimated quality of the average models to the level rarely achievable by a perfect model, but it requires a high validation bias to do so for a bad model. As such, validation bias can arbitrarily inflate model performance estimates. This implies that in any model evaluation result, we cannot be sure if a metric score originates from the real model performance or from validation bias. Coupled with the fact that validation bias is not easily measured, it renders model evaluation metrics obtained using validation sets largely meaningless.

Moreover, it should be noted that validation bias is also not a property of a validation set, but of a pair: the validation set and the prioritization model. Thus, comparisons of different models on the same validation set might not reveal the true relative model performance because performance estimates of different models might be subject to different levels of validation bias, even for the same validation set.

### 3.2 The choice of a metric type influences the ability to detect validation bias

Figure 4 shows that for the average model, AUC ROC and Average Disease Gene Rank detect only the high validation bias (Fig. 4A, B, E and F), while pAUC ROC and recall@300 can also often detect moderate validation bias (Fig. 4C, D, G and H). Similarly, for the bad model, AUC ROC and Average Disease Gene Rank are not always able to detect high validation bias (Fig. 4E and F), while pAUC ROC and recall@300 do so consistently (Fig. 4H–J). Thus, for imperfect models, partial metrics (pAUC ROC, recall@300) do better than the global metrics (AUC ROC, Average Disease Gene Rank).

Considering that *P*-values for recall@*k* and precision@*k* can be obtained analytically without the need for simulations, we strongly suggest utilizing these metrics for detecting validation bias.

For AUC ROC and pAUC ROC the average performance estimates were different than the true performance, which we would get if the validation set contained all disease genes (Fig. 4B, C, F and G). This occurs because the estimates of these metrics depend on the validation set size (Supplementary Materials. Effect of the validation set size on the performance estimation using different metrics, Supplementary Fig. S3).

### 3.3 Validation bias in existing prioritization tools

We used the developed procedure to check the presence of validation bias in the performance evaluation of several previously published gene prioritization tools (Aerts *et al.* 2006, Linghu *et al.* 2009, Ning *et al.* 2015, Isakov *et al.* 2017, Kolosov *et al.* 2021) (Table 1).

**Table 1.** Validation bias testing of papers containing gene prioritization tools evaluations.

Reference	Complex traits	Metric type	Metric value	*P*-value^a^	Minimum assumed number of disease genes for validation bias detection*
Isakov *et al.* (2017)	IBD	recall@150	95/163	<1 × 10^−16^	279
Ning *et al.* (2015)	Crohn’s disease	recall@150	28/54	5.33 × 10^−11^	369
Linghu *et al.* (2009)	Obesity	recall@100	22/334	1.00	2150
Kolosov *et al.* (2021)	IBD	recall@150	20/51	1.21 × 10^−5^	532
	Educational attainment	recall@150	57/564	1.00	1783
	Coronary artery disease	recall@150	20/37	1.69 × 10^−8^	375
	Schizophrenia	recall@150	16/76	8.89 × 10^−2^	1080
Aerts *et al.* (2006)	Atherosclerosis, Crohn’s disease, Parkinson’s disease, rheumatoid arthritis, Alzheimer’s disease	Average disease gene rank	40	<1 × 10^−7^	127

a The lower bound for the number of disease genes for each complex trait is assumed to be 1000 in each test.

* The last column shows the assumed number of genes that allows for validation bias detection (*P*-value <.05). For most of the published validation pipelines, the obtained metrics seem to be too good to be achieved even with a perfect model.

We estimated two indicators of the presence of validation bias: first, the *P*-value under the assumption of the existence of at least 1000 true disease genes for a trait (Section 2.1); second, a minimal number of true disease genes that should exist for the validation bias to be confidently detected in the given settings. As a result, in most applications to specific traits, validation bias was detected (Table 1). This outcome indicates that, in fact, the validation sets were not representative of the entire set of true disease genes, and the estimated performances of gene prioritization tools were inflated.

We proposed that the way we assemble validation sets affects how much we overestimate the performance of gene prioritization tools. Two phenotypes were selected for the analysis—coronary artery disease (CAD), as an example of the phenotype with a decent amount of biological knowledge and genetic association; and schizophrenia—as an example of the trait still lacking comprehensive understanding of the disease biology. In our analysis, we gathered disease genes for schizophrenia using evidence from genome-wide association studies (GWAS) and rare variant association studies (RVAS), while we collected disease genes for CAD solely from GWAS evidence. Separate gene sets were created based on the genetic studies in European and Asian ancestries, from which, population-specific association was extracted.

One gene set from GWAS in European individuals was used to train two gene prioritization models—ToppGene (Chen *et al.* 2009) and GPrior (Kolosov *et al.* 2021). Then, the quality of prioritization for all other assembled gene sets was measured.

Our findings indicated that these tools tend to better prioritize genes derived from other GWAS studies conducted on individuals of European ancestry, compared to those conducted on individuals of Asian ancestry. Additionally, quality of prioritization of the two models was significantly worse for genes obtained from a RVAS (Supplementary Materials, Validation sets assembled using different methods produce different estimations of model performance, Supplementary Figs S4 and S5).

## 4 Discussion

Gene prioritization is an active area of computational biology research. Gene prioritization tools are reported to have great performance but are rarely used in practice, because they yield poor and inconsistent results, as was shown by the Global Biobank Meta-analysis Initiative (Zhou *et al.* 2022). We argue that this difference arises because the usual method of validating gene prioritization using gold standard gene sets leads to overly optimistic performance estimates.

Validation of gene prioritization models and PU-learning algorithms in general rely on the SCAR assumption, which assumes that the probability of being labeled is equal for all positive data points. By leveraging this assumption, one can estimate the performance of these models by treating all unlabeled data as negative. This approach allows us to obtain unbiased estimates of performance metrics like recall@*k* or average disease gene rank as if we had complete knowledge of all the labels.

However, it is worth noting that the SCAR assumption is often assumed for convenience without strong justification. Although it can be challenging to justify this assumption solely based on data, violations of the SCAR assumption can be identified from the data itself. To address this, we proposed a simulation-based approach that only requires a lower bound on the prevalence of positive examples in the data. The lower bound could be approximately calculated given the background information on the disease heritability and the number of already discovered genes. The greater justifiable lower bound corresponds to the greater power to detect the validation bias.

While this lower bound assumption may seem strong, it is significantly less strict compared to other assumptions required for PU validation, such as knowledge about the exact prevalence of positive examples (Claesen *et al.* 2015, Ramola *et al.* 2019). For example, Cancer Gene Census (Sondka *et al.* 2018) encompasses 579 genes with substantial evidence in oncology. Thus, although the exact number of cancer-related genes remains unknown, considering 579 as a conservative lower bound is a secure choice. For some diseases, such a comprehensive list of disease genes might not be available. Then, it might be difficult to establish the lower bound in advance. In these cases, our approach can return the minimum number of total disease genes that are required to reject the SCAR assumption, and then the researcher can decide whether this lower bound is justifiable or not based on available knowledge about the disease.

While it was widely speculated that the SCAR assumption did not hold for gene–disease data due to phenomena such as knowledge bias, it was never confidently established. Through simulations, we showed that validation bias is prevalent in previously published validation pipelines of various gene prioritization methods. Moreover, validation bias has the potential to arbitrarily inflate the estimated performance of models. As such, any performance estimate can be obtained by different combinations of the true model performance and an appropriately biased validation set.

At the same time, standard ranking metrics do not capture the main utility of gene prioritization—the implication of individual genes—and instead evaluate the overall prioritization. All of the above makes the standard approach to the performance estimation of gene prioritization models unrepresentative of the true model utility.

We propose a different approach for the utility assessment of gene prioritization tools. Instead of computing unreliable numerical measures of the overall quality of prioritization, one should report practical findings that were obtained using the model (van Driel *et al.* 2003). For example, for the gene-level association tests used in exome sequencing data analysis, two additional steps are required to justify the robustness of the obtained result—replication in the independent cohort and functional validation, usually involving *in vitro* or *in vivo* experiments.

The illustrated simulation framework can be applied to other PU settings. In particular, the effect of assumptions about the true number of positive data points, validation bias, validation set contamination, and validation set size on performance metric scores can be tested for any PU problem. Additionally, violations of the SCAR assumption can be detected for any PU data for which we can assume a high number of positive data points. Crucially, this framework does not assume anything about the prioritization model itself, making it potentially applicable to any model.

If the analysis shows that the SCAR assumption is violated, it is necessary to use PU-learning techniques that do not rely on this assumption (Bekker *et al.* 2020). Additionally, we advise against using validation sets for model performance estimations, and instead assess the practical utility of a model—specific usage scenarios in which new hypotheses are generated using insights from a model and then validated using experimental techniques. Such an approach does not produce a numerical estimate—thus, it is hard to directly compare different models. However, unless we make additional strong assumptions about how the labeling happens in the PU data and can experimentally validate each highly ranked data point, the comparison is generally difficult. This is because we do not know how validation bias might be affecting the estimated metric values in any particular scenario.

Although the performance estimations obtained using validation sets are not reliable, they can still be used to test the adequacy of the model. It is expected that a model is at least able to prioritize a validation set better than randomly. Thus, a worse-than-random performance would indicate an incorrect model design or implementation.

## Supplementary Material

vbad128_Supplementary_Data

## Data Availability

There is no data associated with our work so there is nothing to deposit.
