# Supplementary material for: Detecting biased validation of predictive models in the positive-unlabeled setting: disease gene prioritization case study
Source: Bioinform Adv. 2023 Sep 14;3(1):vbad128. doi: 10.1093/bioadv/vbad128 (PMC10517638; doi:10.1093/bioadv/vbad128)
Supplement: vbad128_Supplementary_Data [file vbad128_supplementary_data.docx]

**Simulation-based detection of validation bias in positive-unlabeled data with application to disease gene identification.**

**Supplementary Materials**

Ivan Molotkov^1,2,3^, Mykyta Artomov^1,2^

^1^ – Nationwide Children’s Hospital, Columbus, USA

^2^ – The Ohio State University, Columbus, USA

^3^ – ITMO University, Saint Petersburg, Russia

Correspondence: [mykyta.artomov@nationwidechildrens.org](mailto:mykyta.artomov@nationwidechildrens.org)

**The authors declare no conflict of interests.**

**Table of contents:**

| Effect of the validation set contamination with non-disease genes on validation bias detection | 3 |
| --- | --- |
| Effect of the choice of the lower bound on the total disease genes number on validation bias detection | 6 |
| Recall@k is a convenient metric for validation bias detection and prioritization model performance estimation using validation sets | 9 |
| Effect of the validation set size on performance estimation using different metrics | 9 |
| Validation sets assembled using different methods produce different estimations of model performance | 11 |
| Supplementary Tables | 15 |
| References | 18 |

*Effect of the validation set contamination with non-disease genes on validation bias detection*

In the simulations for the validation bias procedure, validation sets were assembled only from the true disease genes. However, in practice, some non-disease genes might end up in the validation sets – these validation sets will be referred to as *contaminated*.

We argue that the assumption of uncontaminated validation sets makes it more difficult to detect validation bias. Thus, if validation bias was detected with this assumption, it would also be detected in the case of the contaminated validation sets. Below, we provide analytical proof and simulations to illustrate the effect of validation set contamination.

**Figure S1** shows the simulated performance estimates of a perfect model using contaminated and uncontaminated validation sets. When accounting for contamination, the estimated performance of the model using all four metrics got worse compared to the uncontaminated validation sets. Thus, if a particular metric value is unattainable with uncontaminated validation sets, it would continue to be so with contaminated ones – validation bias would still be detected.

Moreover, metrics would respond differently to the presence of non-disease genes in validation sets. Performance estimations using global metrics that look at the overall prioritization are influenced significantly more (**Figure S1 A-B**) than those using partial metrics that look at only the top-ranked genes (**Figure S1 C-D**). This happens because for partial metrics contamination means that some True Positive (TP) can become False Positive (FP), while for the global metric it also means that instead of some True Negatives (TN), False Negative (FN) would appear in the lower part of the ranked list. Thus, performance estimations using global metrics should be affected more by validation sets contamination. This means that partial metrics, such as recall@k and pAUC ROC, are more robust to contaminated validation sets.


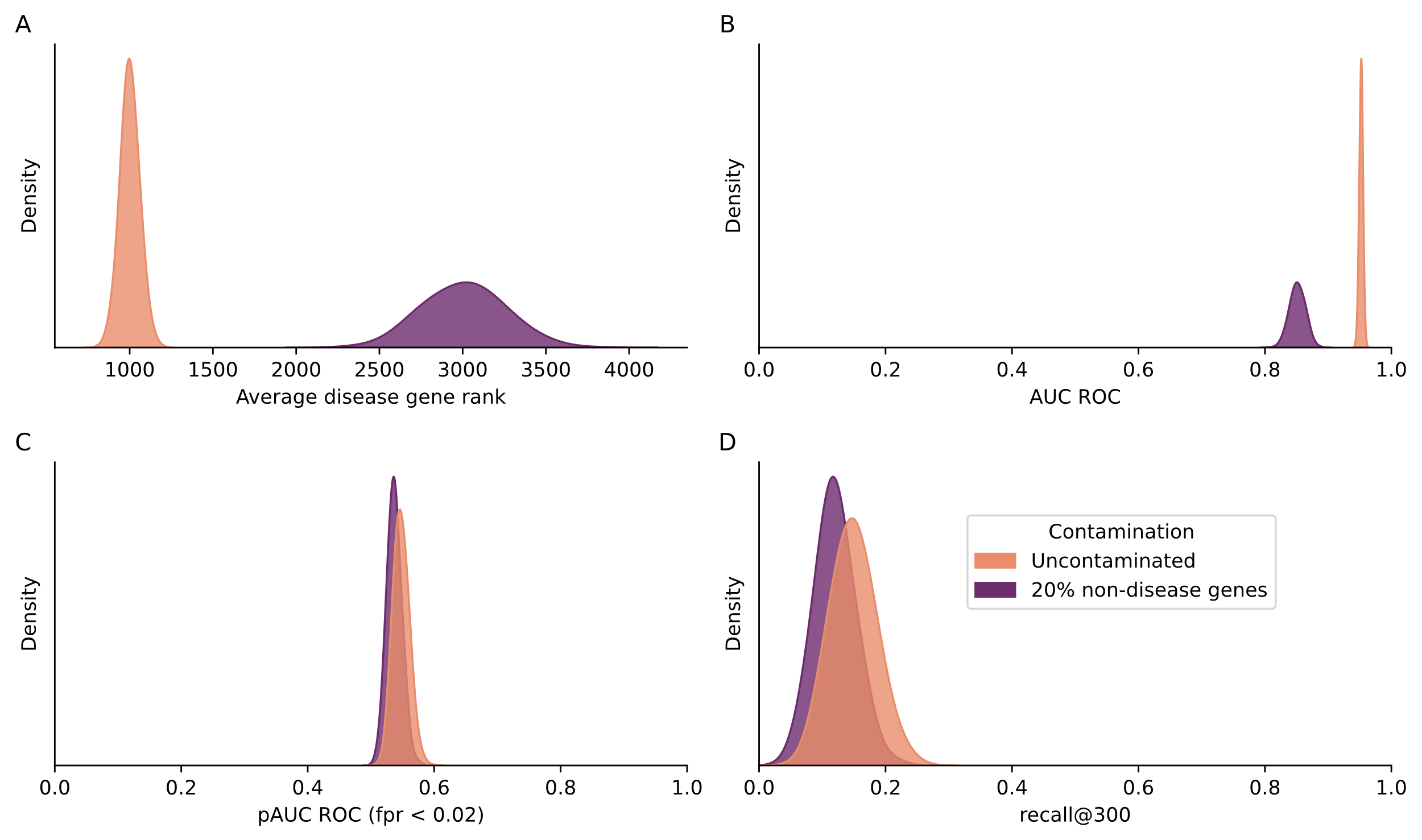


**Figure S1. Effect of the validation set contamination on simulated performance estimates of a perfect model**

A-D: Distributions of simulated metrics of perfect gene prioritization model as estimated from sampled validation sets of size 100 with different levels of non-disease gene contamination.

For each metric, if an estimated model performance value is unattainable for the perfect model with no contamination in the validation sets, then it is still unattainable with contaminated validation sets. However, it might be the case that a combination of an imperfect model and a contaminated validation set can produce a performance estimate that is higher than that of a perfect model and an uncontaminated validation set. If this was the case, unrealistically high performance estimates could arise not from validation bias, but from the contamination in validation sets – and thus, we could not confirm the presence of validation bias. To show that this is not the case, we can investigate which combination of prioritization quality and contamination achieves the best expected metric scores.

We assume that the total number of genes in the genome $M$ and the validation set size $N$ are known. We also assume that only top-$k$ ranked genes are predicted as disease genes. Let’s use the additional notation:

$$M_{d}-number of disease genes$$

$$M_{n}-number of nondisease genes$$

$$n_{d}-number of disease genes in top k ranked genes$$

$$n_{n}-number of nondisease genes in top k ranked genes$$

$$N_{d}-number of disease genes in the validation set$$

$$N_{n}-number of nondisease genes in the validation set$$

Let’s notice that if the number of True Positives (TP), which is the number of genes from the validation set in the top-k ranked ones, is known, we can also derive True Negatives (TN), False Positives (FP) and False Negatives (FN):

$$FN=N-TP$$

$$FP=k-TP$$

$$TN=M-k-N+TP$$

The maximization of TP will also maximize TN while minimizing FP, and FN. This, in turn, results in maximization of the expected estimated model performance. Then, to find out when the expected performance estimate is maximized, it is necessary to investigate what choice of $M_{d},M_{n},n_{d},n_{n},N_{d},N_{n}$ maximizes the expected TP if validation sets are sampled randomly and each contains $N_{d}$ disease genes and $N_{n}$ non-disease genes.

Let’s calculate the separate contribution of disease and non-disease genes to TP count. There are $M_{d}$ disease genes in total, $n_{d}$ of them are in top-$k$. Then, if we sample randomly $N_{d}$, the number of sampled disease genes that are in top-$k$ will have a Hypergeometric($M_{d}, n_{d},N_{d}$) distribution. Similarly, the number of sampled non-disease genes that are in top-$k$ will have a Hypergeometric($M_{n}, n_{n},N_{n}$) distribution. Thus:

$$\mathbb{E}[TP] = \mathbb{E}[Hypergeometric(M_{d}, n_{d},N_{d}) + Hypergeometric(M_{n}, n_{n},N_{n})] == \mathbb{E}[Hypergeometric(M_{d}, n_{d},N_{d})] + \mathbb{E[}Hypergeometric(M_{n}, n_{n},N_{n})]=N_{d}\frac{n_{d}}{M_{d}}+N_{n}\frac{n_{n}}{M_{n}}=N_{d}\frac{n_{d}}{M_{d}}+{(N-N}_{d})\frac{k-n_{d}}{M-M_{d}}\to max$$

To get rid of the constants, we can use scaled variables: proportion of disease gene in validation sets $\tilde{N}_{d}=\frac{N_{d}}{N}\in[0,1]$, proportion of disease genes in top-k ranked genes $\tilde{n}_{d}=\frac{n_{d}}{k}\in[0,1]$ and the proportion of disease genes in all the genes $\tilde{M}_{d}=\frac{M_{d}}{M}\in[0,1]$. Then, we maximize

$$f(\tilde{N}_{d}, \tilde{n}_{d},\tilde{M}_{d})=\tilde{N}_{d}\frac{\tilde{n}_{d}}{\tilde{M}_{d}}+{(1-\tilde{N}}_{d})\frac{1-\tilde{n}_{d}}{1-\tilde{M}_{d}}\to max$$

Without any further constraints, the function can approach infinity by either picking $\tilde{M}_{d}$ close to 0 and $\tilde{N}_{d}, \tilde{n}_{d}$ close to 1, or $1-\tilde{M}_{d}$ close to 0 and $1-\tilde{N}_{d}, 1-\tilde{n}_{d}$ close to 1. If we choose a reasonable constraint on the proportion of disease gene – $\tilde{M}_{d}\in[M_{min},M_{max}]$, where $M_{min}<1-M_{max}$, then there is only one solution that maximizes TP: $\tilde{N}_{d}=1, \tilde{n}_{d}=1,\tilde{M}_{d}=M_{min}$, which corresponds to the assumption of a perfect model; uncontaminated validation sets; the small number of disease genes. Without the $M_{min}<1-M_{max}$ constraint, we get the opposite assumption – the worst model, that ranks disease genes lower than non-disease ones; validation sets contain only non-disease genes; almost all the genes cause the disease.

Thus, it was proven that the assumption of a perfect model and uncontaminated validation sets allows for the highest realistically achievable performance metric values. As such, if those values are still significantly lower than the estimated performance of the tested model – validation bias will be detected no matter whether we allow for contaminated validation sets and imperfect model or not.

Three contributors to the highest realistically achievable performance metric values were identified: validation set contamination, true model performance, and the total number of disease genes. If one can justify lowering the first two or increasing the last one – the power of validation bias detection test would increase, therefore, it would be easier to detect validation bias.

*Effect of the choice of the lower bound on the total disease genes number on the detection of validation bias*

The validation bias detection procedure requires an assumption about the total number of disease genes – $M$. To perform the parameter sensitivity analysis, we investigated how the assumed value of $M$ affects the validation bias detection procedure. To test that, we simulated the distributions of four gene prioritization metrics for a perfect prioritization model with no validation bias (Average Disease Gene Rank, AUC ROC, pAUC ROC (false positive rate < 0.02), recall at 300) for values of $M$ ranging from 500 to 5,000 (**Figure S2. A-D**).

For each $M$, The top 5% of empirical metric values for the perfect model with no validation bias are shown in (**Figure S2. E-H**). These values will be referred to as validation thresholds. If the estimated performance is better than the validation threshold, validation bias is detected.

As $M$ grows, the number of disease genes that are correctly ranked highly but are not in the validation set, and thus treated as false positives (FPs), also increases. Thus, for larger $M$, estimated perfect model performance is supposed to be worse than for smaller $M$ – for example, recall@300 = 0.6 is typical for the $M$ = 500 case but is unattainable in the $M$ = 5000 case.

However, the choice of $M$ influences the validation thresholds of different metrics unequally. For example, when $M$ is increased from 500 to 5000, the AUC ROC validation threshold changes moderately - from 0.99 to 0.89, but the recall@300 validation threshold changes dramatically - from 0.65 to 0.1. Thus, if we can justify the assumption of a high number of disease genes, a metric like recall@k would be better at validation bias detection.

*
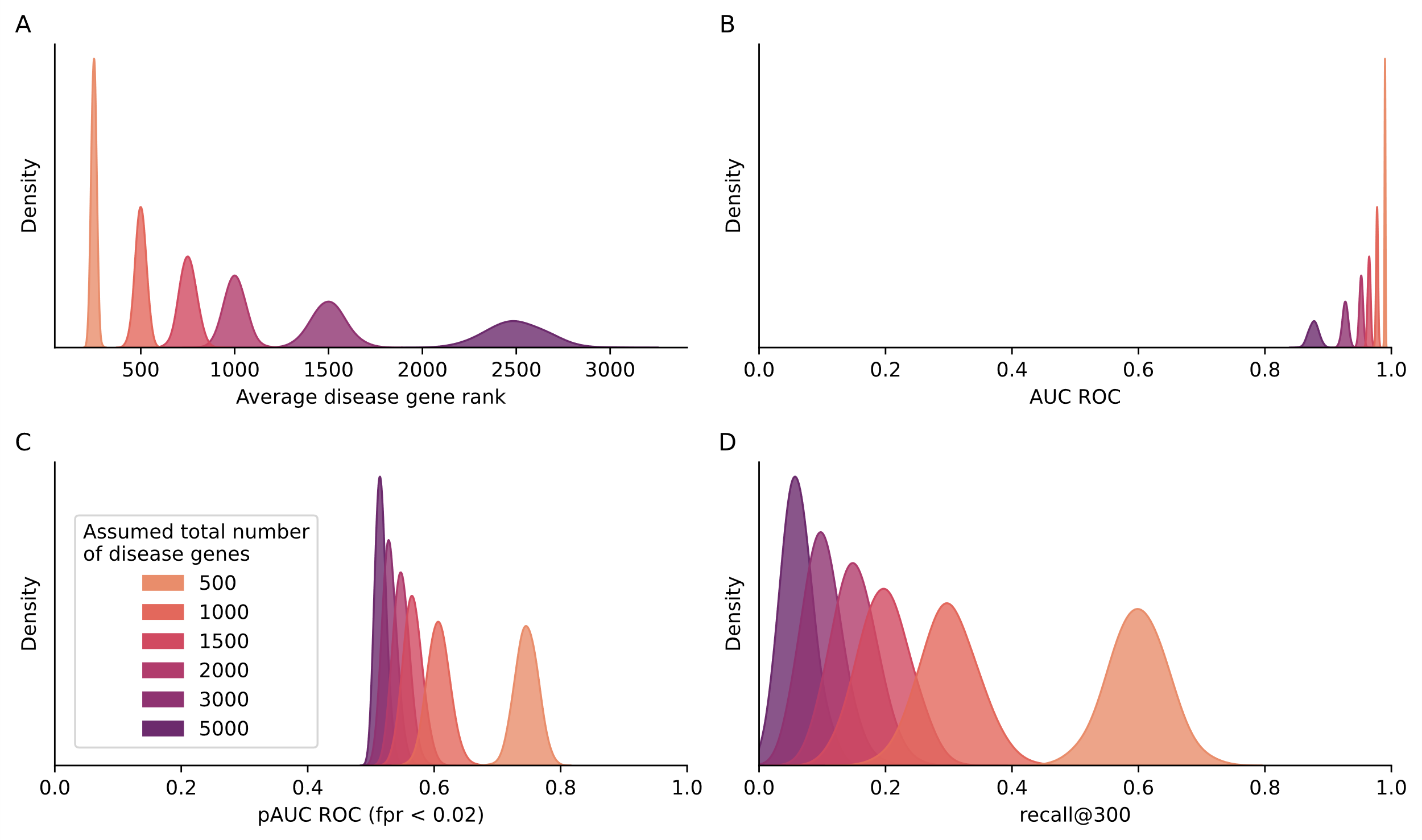
*

*
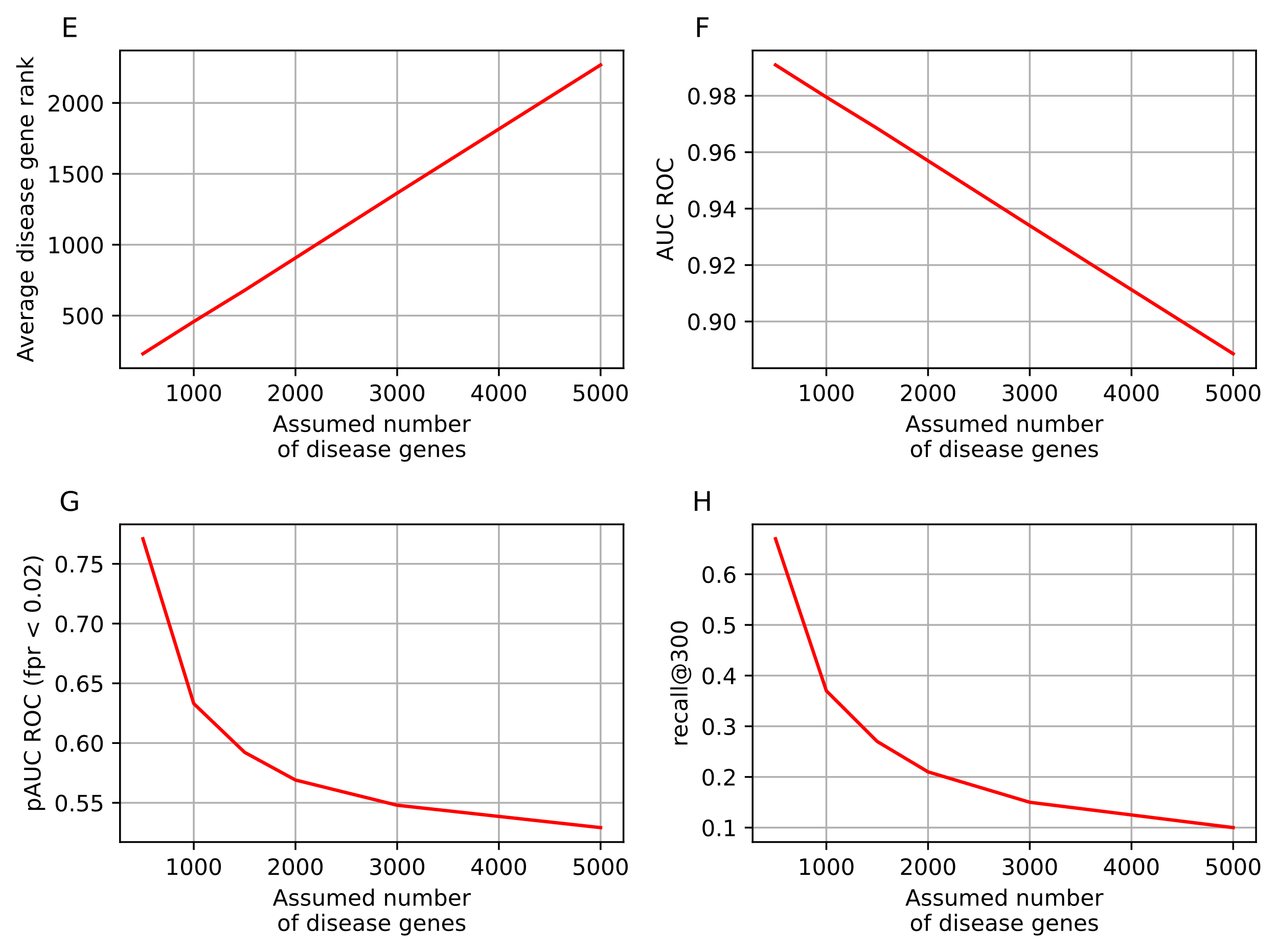
*

**Figure S2. Effect of the choice of the lower bound on the total disease genes number on the detection of validation bias.**

A-D: Distributions of evaluation metric values for simulated models as estimated from sampled validation sets of size 100 for different assumed total numbers of disease genes.

E-H: Simulated validation thresholds for the same metrics for different assumed total numbers of disease genes.

*Recall@k is a convenient metric for validation bias detection and prioritization model performance estimation using validation sets*

In the process of gene prioritization model validation using validation gene sets, the choice of prioritization performance estimation metrics should serve two purposes: (i) validation bias should be easily detectable when present using this metric and (ii) in the absence of validation bias, estimated performance in expectation should be equal to the true performance metric value that we would obtain if all the disease genes were known.

We propose recall@k as a metric that has all the desired properties listed above. It showed a good ability to detect validation bias (**Results. Choice of a metric type influences the ability to detect validation bias**), recall@k validation thresholds can be computed analytically, and estimates obtained using validation sets for this metric in expectation are equal to the true recall@k.

Recall@k represents the proportion of genes from the validation set that are in the list of top-k ranked genes: $recall@k=\frac{TP}{TP+FN}=\frac{TP}{Validation set size}=TPR$, if we consider only top-k ranked genes to be positive predictions. For this value, only the numerator is random and depends on a particular validation set that is used for the estimation.

Under the assumption of the perfect model, no validation bias and known number of disease genes, the generative process of TP values can be described as follows: (i) there are $M$ disease genes in total, (ii) k of them are top-k ranked because we use the perfect model, (iii) $N$ of the disease genes are sampled into a validation set. Then, TP indicates how many of the sampled genes are in the top-k. Using this formulation, we can notice that the number of TPs follows a hypergeometric distribution with total population of $M$; k objects of interest and $N$ draws.

Therefore, we can use quantiles of the hypergeometric distribution to produce validation thresholds. Also, since the mean of the hypergeometric distribution is $E\left( TP \right)=N\frac{k}{M}$, we have that $E\left( recall@k \right)=\frac{1}{N}E\left( TP \right)=\frac{k}{M}$ does not depend on the validation set size, unlike FPR-based metrics like AUC ROC as discussed in (**Supplementary Materials. Effect of the validation set size on the performance estimation using different metrics**).

As such, recall@k is efficient at validation bias detection (**Figure 4E,J**), does not require simulations and its estimate using validation sets is equal in expectation to the true metric value and does not depend on the validation set size.

*Effect of the validation set size on the performance estimation using different metrics*

When analyzing a validation pipeline, validation set size is known in advance -therefore, no assumptions about it are necessary. However, it can still be informative to test how model performance estimates for different metrics respond to the choice of the validation set size.

**Figure S3** illustrates distributions of performance estimates for the perfect model using sampled validation sets of sizes ranging from 20 to 500.

For all the metrics, variance of estimates decreased as the validation set size increased. For Average Disease Gene Rank and recall@k, the distribution mean did not change, and was equal to the true performance of the model that would be obtained if all the disease genes were known (**Figure S3 A,D**). However, the expected estimates of AUC ROC and pAUC ROC increased along with the validation set size (**Figure S3 B,C**). As such, even in the absence of validation bias, the expected estimates of those metric would still be unrepresentative of the true performance and would depend on the validation set size.

The dependence of the expected estimate on the validation set size is the undesirable property of metrics that are based on the False Positive Rate (FPR). Model evaluation using validation sets implies a high number of spurious FPs – disease genes that are correctly identified as such by the model but are labeled as non-disease in the data, because they were not selected to the validation set. Therefore, in the absence of validation bias, FPR-dependent method will systematically underestimate the true performance. Moreover, the scale of underestimation will itself be dependent on the validation set size. Thus, to improve the quality of performance estimate, one could simply add more data to the validation set (**Figure S3 B,C**). This fact strongly influences the comparison between the models, as the size of the validation sets that were used to estimate the performance would affect the outcome of the comparison.


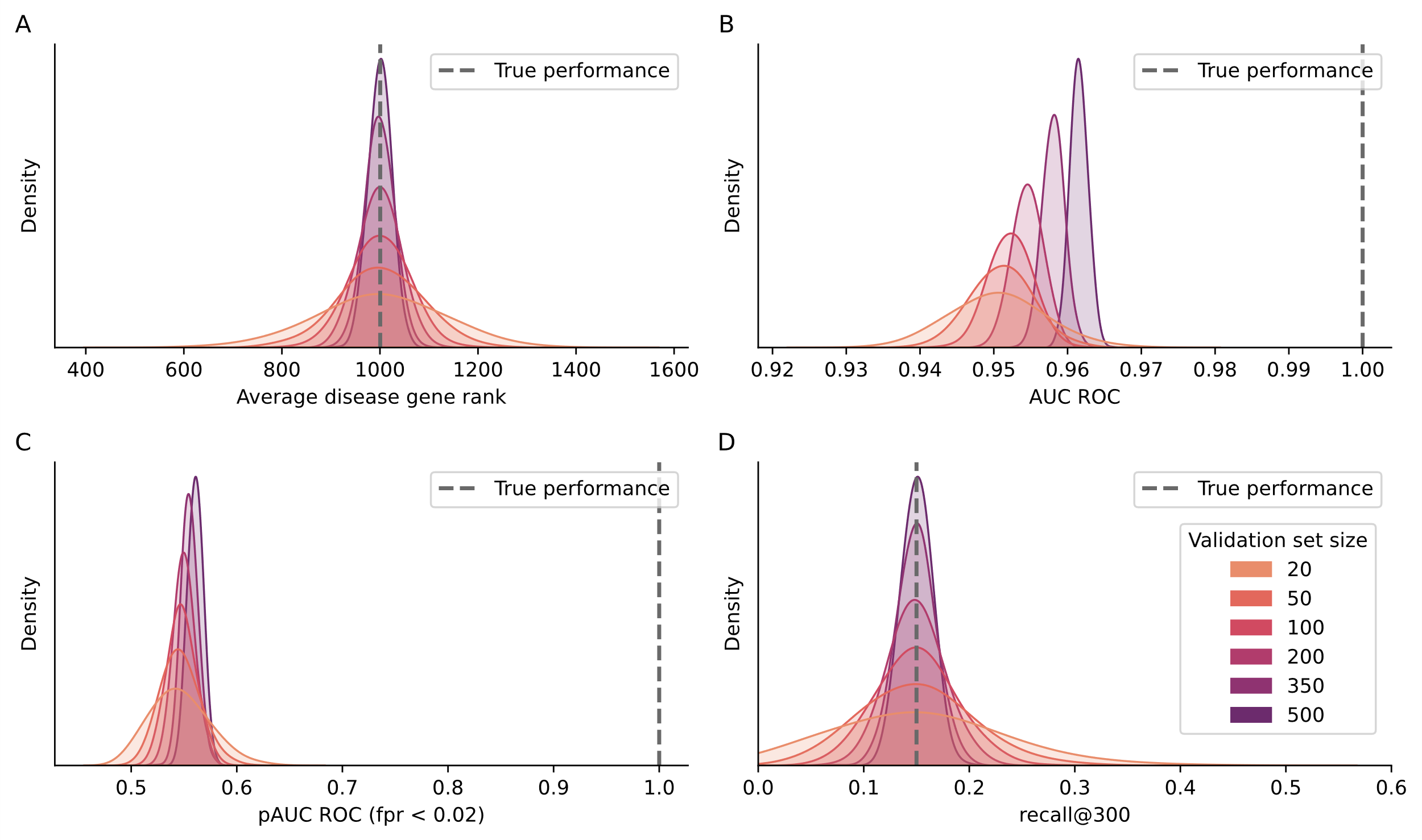


**Figure S3. Effect of the validation set size on the performance estimation using different metrics.**

A-D: Distributions of performance evaluation of a simulated model as estimated from sampled validation sets of sizes ranging from 20 to 500 using different metrics.

*Validation sets assembled using different methods produce different estimations of model performance*

We tested how alternative approaches to validation set assembly can influence model performance estimates. Using different validation sets, performance was estimated for two gene prioritization tools with immediately available prioritization results for several phenotypes - ToppGene^1^ and GPrior^2^. In addition, we used population-specific GWAS association to assemble the validation sets.

The GPrior prioritization results were taken from the supplementary materials of the original paper^2^. The web version [toppgene.cchmc.org/prioritization.jsp](https://toppgene.cchmc.org/prioritization.jsp%20) of ToppGene was used with all features and default parameters for prediction. Training sets for both tools were obtained from supplementary material of the original GPrior paper (**Supplementary Table 1**).

For all phenotypes, a lower bound of 1000 disease genes total was chosen for the validation bias detection procedure.

Experiment 1:

Four different validation sets for Schizophrenia were assembled: two were obtained from two European GWAS^3,4^, one from Asian GWAS^5^, one from European RVAS^6^ (**Supplementary Table 2**).

The European GWAS significantly overestimated the performance compared to the Asian GWAS and the European RVAS (**Figure S4 A-B**). Moreover, in the GPrior’s cases validation bias was detected for all the GWAS validation sets (**Figure S5 A,C,D**). In the case of ToppGene, validation bias was detected for a European GWAS validation set (**Figure S5 C**).

Experiment 2:

Lists of genes associated with Coronary Artery Disease (CAD) [ebi.ac.uk/gwas/efotraits/EFO_0001645](https://www.ebi.ac.uk/gwas/efotraits/EFO_0001645) and Schizophrenia (SCZ) [ebi.ac.uk/gwas/efotraits/MONDO_0005090](https://www.ebi.ac.uk/gwas/efotraits/MONDO_0005090) were obtained from GWAS Catalogue. Only genes for which there was unambiguous ethnicity of patients and p-value < 5x10^-10^ were used. Genes, that were found in both the Asian and the European validation sets, were removed. Full lists of validation genes are in **Supplementary Table 3.**

In all four comparisons, European-specific genes from GWAS were ranked higher than Asian-specific genes from GWAS (**Figure S4 C-F**). Validation bias was detected for European GWAS in GPrior for both phenotypes (**Figure S5 F,H**). In the case of ToppGene, validation bias was not detected for any validation set – however, if the assumptions of only 1000 disease genes or a perfect model were relaxed, validation bias would be found.

It is important to note that since the estimated performance is composed of the true model performance and validation bias, we cannot know whether the fact that validation bias was detected for one tool, and not the other, indicates the better true performance of the former or the lesser validation bias of the latter.


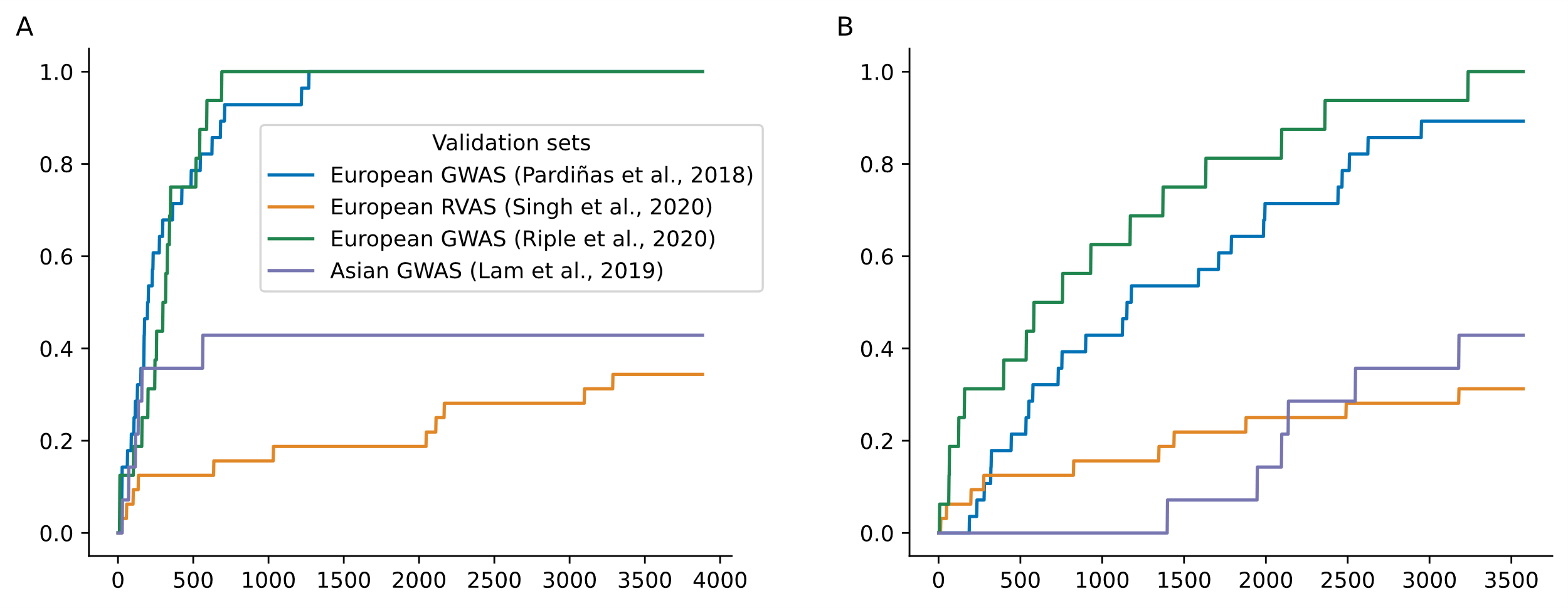


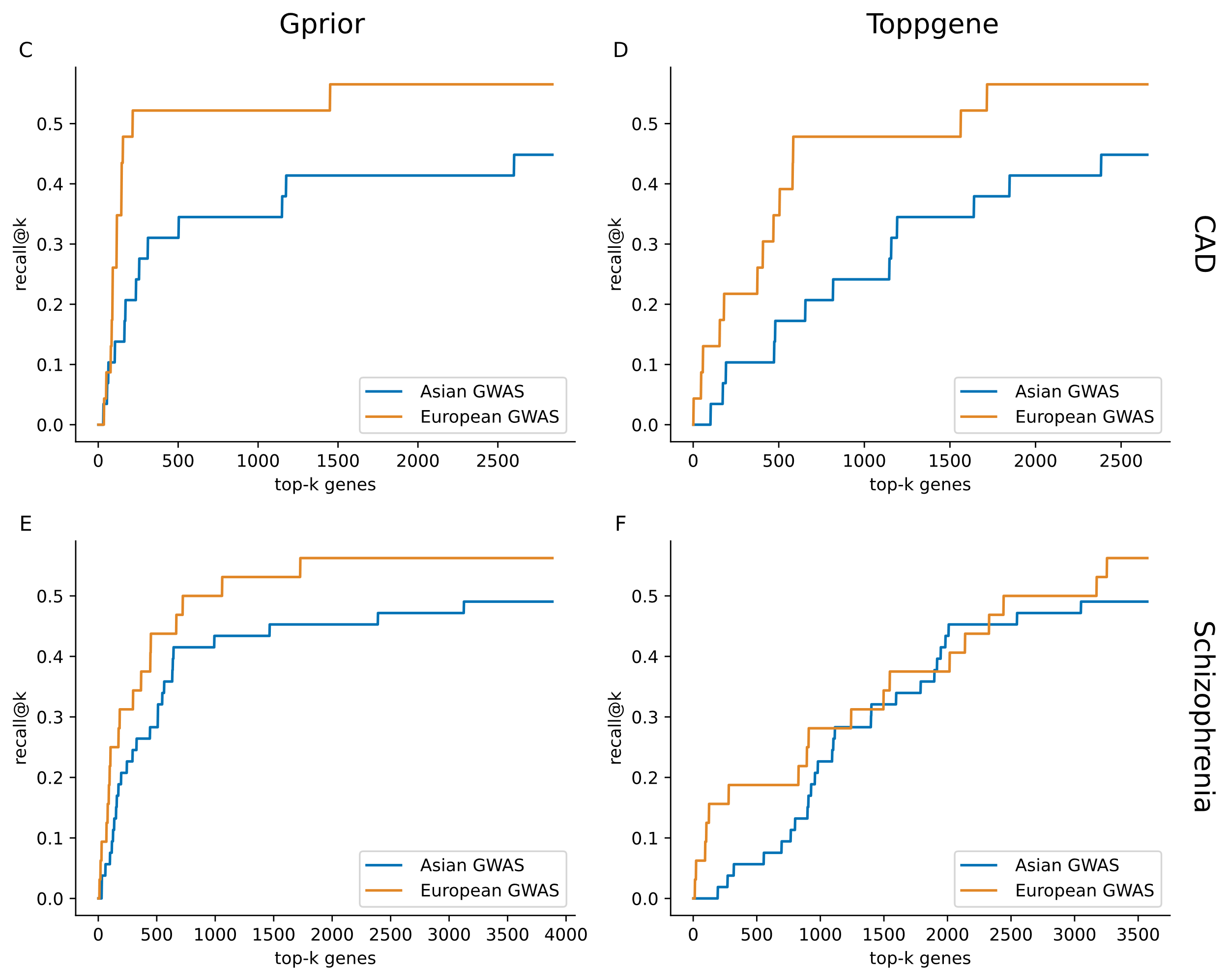


**Figure S4. Performance comparison of two gene prioritization tools on validation sets obtained from different ethnicities and by different procedures.**

A-B: ToppGene and GPrior were evaluated on four different validation sets of schizophrenia-related genes. The evaluated performance significantly drops when RWAS or Asian GWAS are used to form validation sets.

C-F: The performance of GPrior and ToppGene was tested on Schizophrenia (SCZ) and Coronary Artery Disease (CAD) using validation sets obtained from GWAS Catalog from European and Asian ethnicities separately. Model evaluation metric was consistently lower for Asian-specific genes from GWAS.


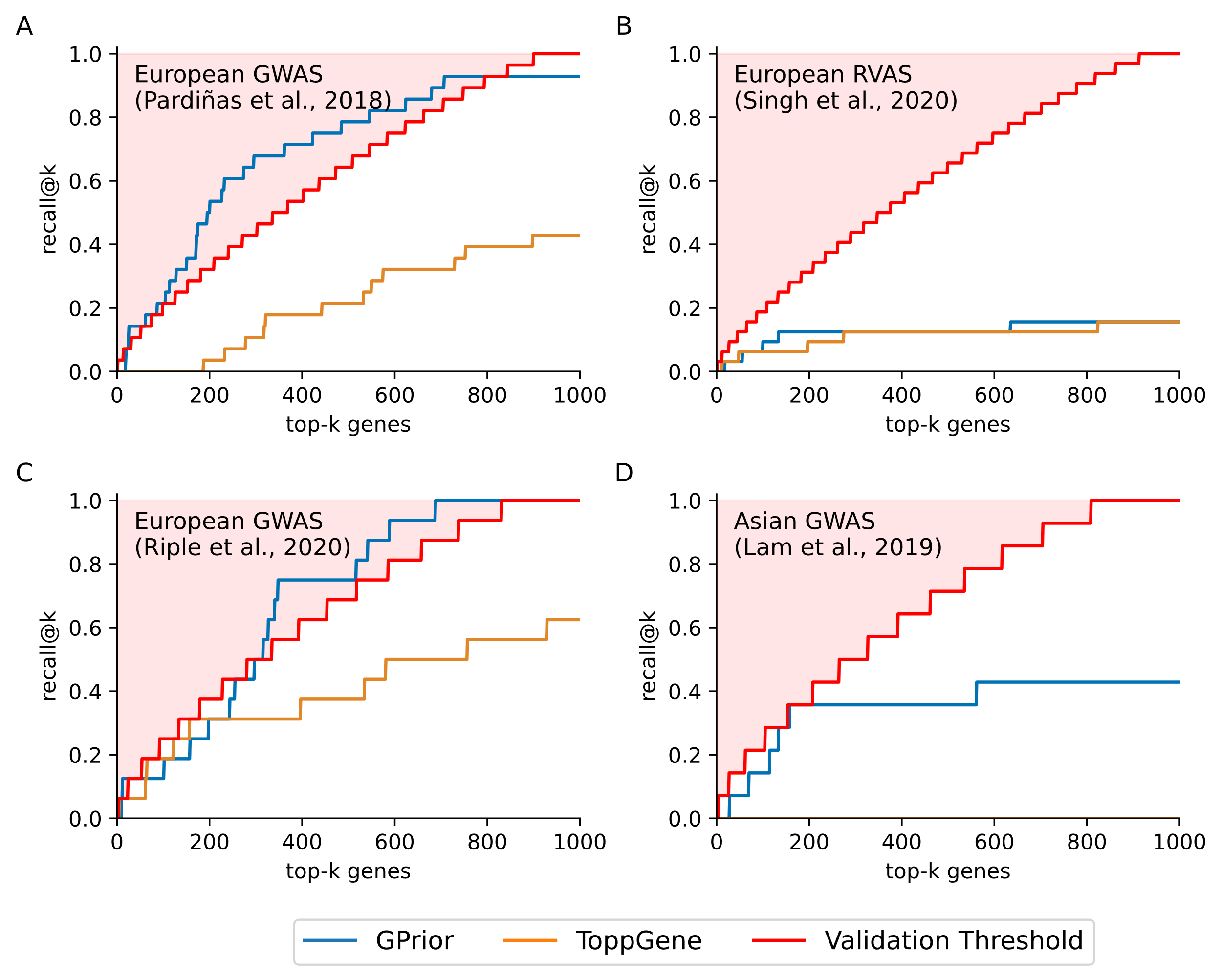


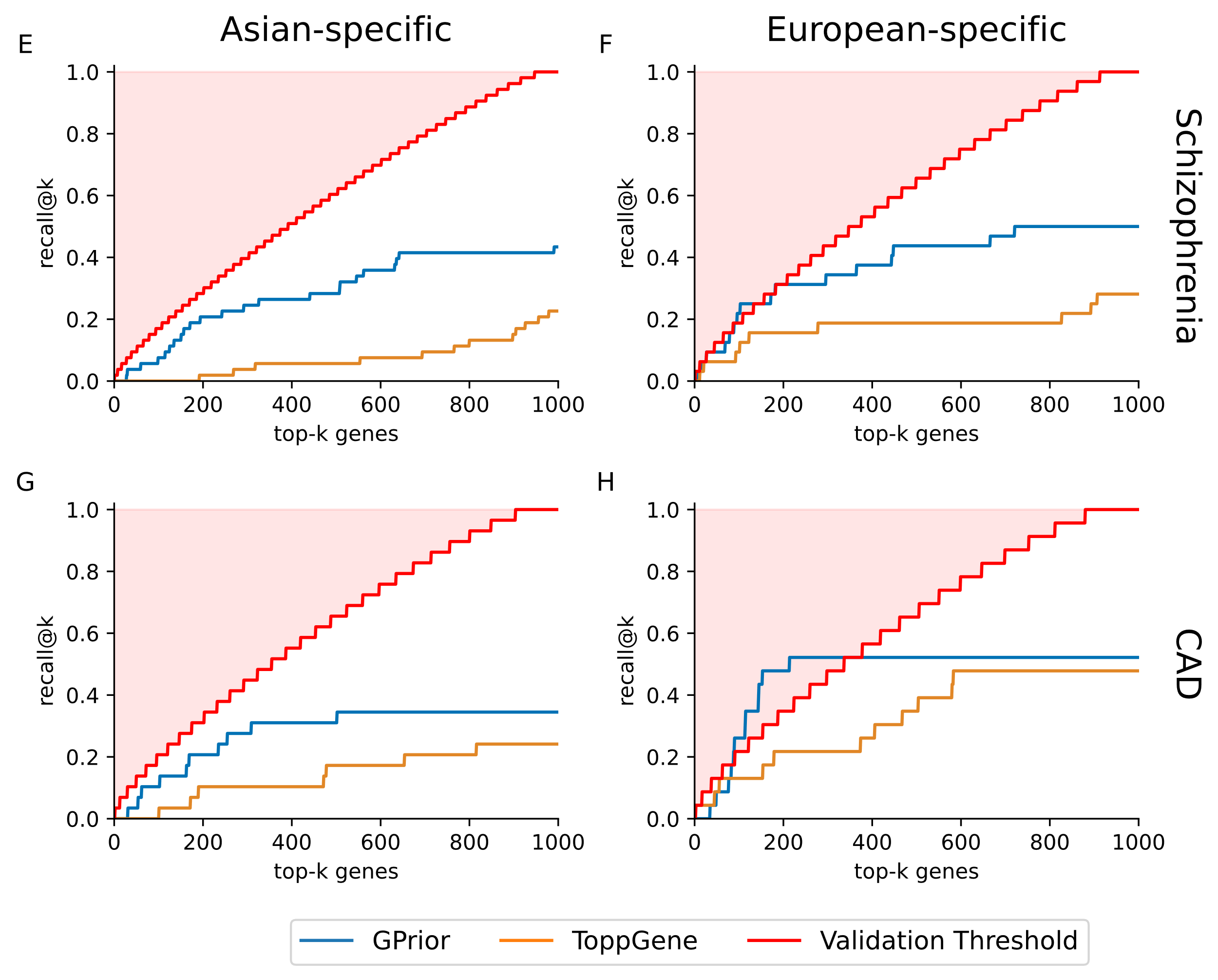


**Figure S5. Comparison of the estimated performance of GPrior and ToppGene with validation thresholds (at least 1000 disease genes assumed).**

Quality of GPrior and ToppGene was estimated using validation sets from **Supplementary table 2 (A-D) and Supplementary table 3 (E-H)**. The obtained estimates were compared with validation thresholds. Red area represents detection of validation bias.

**Supplementary Table 1.** Training gene sets for Schizophrenia and Coronary Artery Disease (CAD)

| **Schizophrenia** | **Coronary Artery Disease** |
| --- | --- |
| CACNA1C | MIA3 |
| TSNARE1 | LDLR |
| SLC39A8 | CXCL12 |
| MAD1L1 | EDNRA |
| ZSWIM6 | APOB |
| TRANK1 | APOA5 |
| IMMP2L | PCSK9 |
| SNX19 | BCAS3 |
| ZNF804A | IL6R |
| CACNB2 | ZC3HC1 |
| LRRIQ3 | LIPA |
| KCNV1 | SWAP70 |
| CNTN4 | ADAMTS7 |
| DRD2 | LPA |
| IGSF9B | ABCG8 |
| GRM3 | ABCG5 |
| SNAP91 |  |
| PLCH2 |  |
| ERCC4 |  |
| ATP2A2 |  |

**Supplementary Table 2.** Curated validation sets for schizophrenia.

| **European GWAS (Pardiñas et al., 2018)** | **European RVAS (Singh et al., 2020)** | **European GWAS (Riple et al., 2020)** | **Asian GWAS (Lam et al., 2019)** |
| --- | --- | --- | --- |
| DPYD | SETD1A | PCDH17 | VRK2 |
| ARL3 | CUL1 | ZNF281 | SPATS2L |
| ABCB9 | XPO7 | PTBP2 | RASSF1 |
| FES | TRIO | WSCD2 | DOCK3 |
| LRP1 | CACNA1G | TRPC4 | STAG1 |
| C2orf82 | SP4 | KLF10 | SOX2-OT |
| ESAM | GRIA3 | NEGR1 | MIR124-2HG |
| AMBRA1 | GRIN2A | ZNF365 | WBP1L |
| CCDC39 | HERC1 | BCL11A | WSCD2 |
| FANCL | RB1CC1 | CALN1 | PITPNM2 |
| ADAMTSL3 | ASH1L | GALNT2 | FRY |
| ANKRD44 | SV2A | CXXC4 | YWHAE |
| GLT8D1 | DNM3 | PPARGC1A | KCNG2 |
| ALDOA | STAG1 | NMUR2 | SLC66A2 |
| MSL2 | NR3C2 | OLA1 |  |
| KDM4A | HIST1H1E | SORCS3 |  |
| ANP32E | PREP |  |  |
| RERE | MAGI2 |  |  |
| C4orf27 | FAM120A |  |  |
| BTBD18 | FAM178A |  |  |
| DGKI | QRP4 |  |  |
| AKT3 | DAGLA |  |  |
| ANKRD63 | SLC22A11 |  |  |
| CDC25C | AKAP11 |  |  |
| ATXN7 | ZMYM2 |  |  |
| CLU | SRRM2 |  |  |
| DFNA5 | HCN4 |  |  |
| DPP4 | KDM6B |  |  |
|  | ANKRD12 |  |  |
|  | ZNF136 |  |  |
|  | EIF2S3 |  |  |
|  | MAGEC1 |  |  |

**Supplementary Table 3.** Validation sets for Schizophrenia and CAD from GWAS Catalog

| **Schizophrenia: Asian GWAS** | **Schizophrenia: European GWAS** | **Coronary Artery Disease: Asian GWAS** | **Coronary Artery Disease: European GWAS** |
| --- | --- | --- | --- |
| COX11P1 | RNU7-147P | ALDH2 | MRAS |
| HFE | LINC00637 | MYL2 | PLPP3 |
| LINC01648 | BTN3A1 | MUC22 | GGCX |
| MTHFR | LINC01470 | COL6A3 | MORF4L1 |
| PITPNM2 | NT5C2 | CISD1P1 | SMAD3 |
| SYNGAP1 | SCAF1 | TMOD4 | RHOA |
| ZDHHC2 | RIMS1 | COL4A2 | APOE |
| TNXB | ATXN7 | PLCB2 | SMARCA4 |
| HLA-DQB1 | ZNF536 | CUX2 | TRIM65 |
| SLC17A4 | NDRG4 | THSD7A | DENND2B |
| FOXO3 | RGS6 | FGD6 | JCAD |
| PBRM1 | PGBD1 | DAB1 | ATXN2 |
| PES1 | SGCZ | CNNM2 | LINC00841 |
| LINC02057 | GPM6A | CTNNA2 | NAA25 |
| CUL3 | GULOP | BET1L | TCF21 |
| BNIP3L | FXR1 | TSBP1-AS1 | ZPR1 |
| LINC02829 | NLGN4X | NUTF2P8 | NOS3 |
| TSPAN18 | ITIH1 | VPS33B | LINC02881 |
| RASSF1 | GRIN2A | ZNF77 | HHIPL1 |
| LSM1 | R3HDM2 | ADGRL3 | CELSR2 |
| UBE2Q2P1 | LINC01929 | FLT1 | FTO |
| SLC66A2 | TMEM219 | DOCK6 | TENT5A |
| SATB2 | PJA1 | TMEM91 | FLJ40194 |
| LINC01149 | MIR137HG | SPC24 |  |
| AMBRA1 | SF3B1 | DAB2IP |  |
| MDK | MOG | ACAD10 |  |
| SPATS2L | LINC01360 | CXCL8 |  |
| HYKK | GGNBP1 | HECTD4 |  |
| DPYD | MEF2C-AS1 | LINC02732 |  |
| GRAMD1B | DGKI |  |  |
| SOX2-OT | GIGYF2 |  |  |
| NKAPL | HCN1 |  |  |
| STAU2 |  |  |  |
| ZBED9 |  |  |  |
| H2BP5 |  |  |  |
| BTN1A1P1 |  |  |  |
| ZKSCAN4 |  |  |  |
| POM121L2 |  |  |  |
| LETM2 |  |  |  |
| SRPK2 |  |  |  |
| SINHCAFP3 |  |  |  |
| BORCS7-ASMT |  |  |  |
| LINC02551 |  |  |  |
| DDX10P2 |  |  |  |
| WBP1L |  |  |  |
| LINC00240 |  |  |  |
| TRIM27 |  |  |  |
| FLOT1 |  |  |  |
| H2AC14 |  |  |  |
| ADAMTSL3 |  |  |  |
| PSORS1C1 |  |  |  |
| SNORC |  |  |  |
| SPHKAP |  |  |  |

**References**

1. Chen, J., Bardes, E. E., Aronow, B. J. & Jegga, A. G. ToppGene Suite for gene list enrichment analysis and candidate gene prioritization. *Nucleic Acids Res* **37**, (2009).

2. Kolosov, N., Daly, M. J. & Artomov, M. Prioritization of disease genes from GWAS using ensemble-based positive-unlabeled learning. *European Journal of Human Genetics* **29**, 1527–1535 (2021).

3. Ripke S, Walters JTR & O’Donovan MC. Mapping genomic loci prioritises genes and implicates synaptic biology in schizophrenia. *medRxiv* (2020) doi:10.1101/2020.09.12.20192922.

4. Pardiñas, A. F. *et al.* Common schizophrenia alleles are enriched in mutation-intolerant genes and in regions under strong background selection. *Nat Genet* **50**, 381–389 (2018).

5. Lam, M. *et al.* Comparative genetic architectures of schizophrenia in East Asian and European populations. *Nat Genet* **51**, 1670–1678 (2019).

6. Singh, T. *et al.* Rare coding variants in ten genes confer substantial risk for schizophrenia. *Nature* **604**, 1–9 (2022).
